# Nano-Vesicle Based Anti-Fungal Formulation Shows Higher Stability, Skin Diffusion, Biosafety and Anti-Fungal Efficacy In Vitro

**DOI:** 10.3390/pharmaceutics12060516

**Published:** 2020-06-05

**Authors:** Isaac G. Deaguero, Md Nurul Huda, Victor Rodriguez, Jade Zicari, Taslim A. Al-Hilal, Abu Zayed Md Badruddoza, Md Nurunnabi

**Affiliations:** 1 Biomedical Engineering Program, School of Engineering, University of Texas at El Paso, TX 79902, USA; igdeaguero@miners.utep.edu (I.G.D.); mdhuda@miners.utep.edu (M.N.H.); varodriguez2015@gmail.com (V.R.); jezicari@miners.utep.edu (J.Z.); taalhilal@utep.edu (T.A.A.-H.); 2 Department of Pharmaceutical Sciences, School of Pharmacy, University of Texas at El Paso, TX 79902, USA; 3 Department of Chemical and Life Sciences Engineering, Virginia Commonwealth University, Richmond, VA 23284, USA; 4 Border Biomedical Research Center, University of Texas at El Paso, TX 79902, USA; 5 Department of Environmental Science and Engineering, University of Texas at El Paso, TX 79902, USA

**Keywords:** miconazole, nano-vesicle, emulsion, anti-fungal, transdermal drug delivery

## Abstract

Opportunistic fungal infections are responsible for over 1.5 million deaths per year. This has created a need for highly effective antifungal medication to be as potent as possible. In this study, we improved the efficacy of a common over the counter (OTC) antifungal skin medication, miconazole, by encapsulating nano-molecules of the drug in cholesterol/sodium oleate nano-vesicles. These nano-vesicles were characterized to optimize their size, zeta potential, polydispersity index and encapsulation efficiency. Furthermore, these nano-vesicles were compared to a conventional miconazole-based commercially available cream to determine potential improvements via permeation through the stratum corneum, cytotoxicity, and antifungal capabilities. Our results found that the vesicle size was within the nano range (~300 nm), with moderate polydispersity and stability. When compared with the commercially available cream, Actavis, as well as free miconazole, the miconazole nano-vesicle formulation displayed enhanced fungal inhibition by a factor of three or more when compared to free miconazole. Furthermore, with smaller nanoparticle (NP) sizes, higher percentages of miconazole may be delivered, further enhancing the efficacy of miconazole’s antifungal capability. Cytotoxicity studies conducted with human dermal fibroblast cells confirm its biosafety and biocompatibility, as cell survival rate was observed to be twofold higher in nano-vesicle formulation than free miconazole. This formulation has the potential to treat fungal infections through increasing the retention time in the skin, improving the treatment approach, and by enhancing the efficacy via the use of nano-vesicles.

## 1. Introduction

Globally, 300 million people are inflicted with a serious fungal infection annually [1]. Of those infected, 25 million will face the possibility of losing their sight [1]. Fungal infections are the cause of over 1.5 million deaths per year [1]. Most of these deaths are caused by opportunistic infections acting on already immunocompromised individuals, such as those diagnosed with HIV/AIDS, influenza, cancer, Chronic obstructive pulmonary disease (COPD), asthma, tuberculosis, or those currently taking immunosuppressants for a variety of medical reasons [2]. Essentially, these fungal infections prey on the most vulnerable in society and, therefore, a high degree of significance must be placed on ensuring that current therapies are achieving maximum efficacy.

Tinea Corporis, a dermatophyte commonly known as ringworm, is one of these opportunistic infections that plagues many individuals, especially the immunocompromised [3].This type of disease subsides deep within the skin, using these layers as a barrier to protect itself from external threats, while it manifests and spreads beneath the stratum corneum, wreaking havoc on the body and, if left untreated in an immunocompromised adult, may eventually lead to serious illness or death [4]. Tinea Corporis is a pathogenic fungus that is often found on all areas of mammalian skin, and can be seen on the surface layers, often creating a distinctive ring pattern, and is especially prominent on those with weakened immune systems, such as those infected with HIV/AIDS [5].

Superficial skin infections are often difficult to treat and require the use of systemically dispersed drugs to properly attack the site of infection [6]. This is primarily due to the makeup of the skin, which consists of a protective outer layer of keratin and lipids known as the stratum corneum (SC) layer. This layer is the primary defense against foreign invaders and allows very few molecules to pass through into the deeper layers of the skin [7]. Due to its protective nature, passing foreign material through this layer is quite challenging, and requires a specific size and outer makeup to pass to the lower layers of skin [8].

Miconazole is an imidazole antifungal agent, commonly used to treat various fungal infections of the skin and vagina [9]. It is available in tablet, cream, ointment, and suppository form, and may also be delivered intravenously in some circumstances. This drug is currently listed on the World Health Organization’s list of essential medicines: those which are considered to be the most effective and safe medicines necessary to meet the major and minor needs of a health system [10]. This drug is also extremely affordable, being one of the most cost-effective antifungal agents available over the counter, and available in virtually every country worldwide [11]. Miconazole is not considered toxic due to its high Lethal Dose 50% (LD50) and is especially attractive due to its low half maximum effective concentration (EC50) [11]. However, miconazole is water-insoluble, making it difficult to deliver to the target area in large quantities beneath the skin [12].

This glaring issue involving miconazole has led to most creams and conventional treatments to be limited to containing 1–2% of the drug with an abundance of lipid fillers to transport the drug to the target site [13]. On average, for a typical ringworm infection, miconazole cream must be applied twice daily, for 4 weeks to eliminate the infection [9]. This means that a conventional treatment requires almost 60 applications over the course of a month. Due to this issue, patient compliance is low and leads to recurring infections that may last years in an otherwise healthy person. Continuous manifestation may lead to scarring at the site of infection and can both spread and infect new hosts during this time.

**Figure 1:**
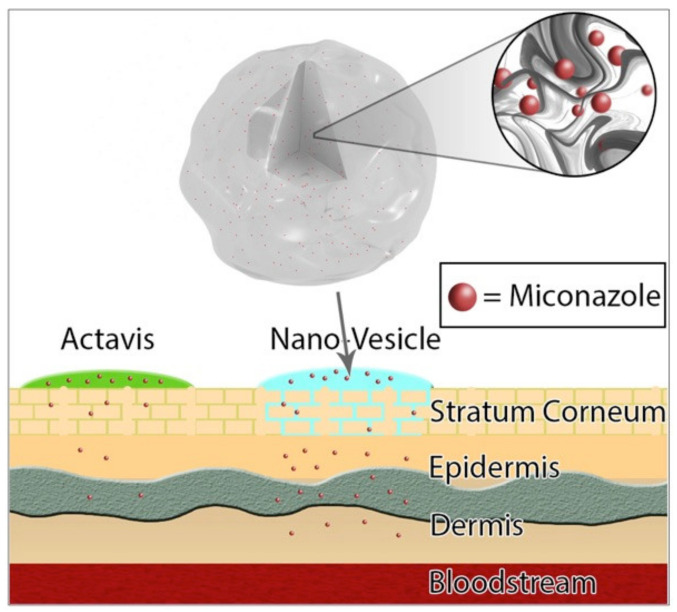
Miconazole loaded nano-vesicles offer a higher delivery of drug content through the epidermis to the target site of fungal infection. The high lipid content of the nano-vesicles allows the drug to be carried through the stratum corneum and offers a previously unattainable avenue of drug delivery in high content for lipophilic nano-sized drugs.

This study attempts to remedy this plethora of issues by securing adequate transport of miconazole to the site of infection, while still involving a familiar route of skin entry through conventional transdermal skin application (Figure 1). Lipid encapsulation has been shown to enhance delivery while also increasing the efficacy of antifungal drugs [14]. Previous studies involving the use of a combination of cholesterol and sodium oleate with proper ratios have been successful in transdermal drug delivery, while maintaining respectable drug encapsulation efficiency [15]. Understanding the proper ratios of miconazole, cholesterol, and sodium oleate, as well as measuring vesicle size, polydispersity index, zeta potential, and encapsulation efficiency are paramount to understanding overall drug efficacy, and were intimately studied to ensure that the best combination to battle infection would be properly utilized for further study.

Additionally, proper measurement through the skin layers was of prime importance [16], especially when compared to Actavis creams, and across a spectrum of in vitro human skin types, to ensure result consistency [17]. In vitro cytotoxicity and efficacy against the dermal cell line and potential fungi, respectively [18], was also required to showcase the efficacy of lipid-enhanced miconazole nanoparticles when compared to free miconazole.

## 2. Materials and Methods

### 2.1. Materials

Miconazole was purchased from MP Biomedicals (Solon, OH, USA). Actavis (miconazole 2%) was purchased from a local pharmacy store in El Paso, TX, USA. Cholesterol was purchased from Alfa Aesar (Ward Hill, MA, USA). Cadaver skin for permeation and skin diffusion studies was obtained from Zen-Bio Inc. (Research Triangle Park, NC, USA). Deionized water (resistivity of 18.2 MΩ) used for all experiments was obtained from an in-house Milli-Q^®^ IQ 7000 Ultrapure Water System (EMD Millipore, Bedford, MA, USA). Sodium oleate was purchased from TCI America (Portland, OR, USA). Methanol (ACS grade), phosphate buffer saline (PBS, pH 7.4), and chloroform were procured from Fisher Chemicals (Fair Lawn, NJ, USA).

### 2.2. Preparation of Nano-Vesicles

Miconazole loaded nano-vesicles were prepared by using a thin-film hydration method previously used in a similar azole study [15]. Miconazole, cholesterol, and sodium oleate were dissolved in 10 mL of methanol and chloroform in a 1:1 ratio solution. This solution was placed in a 15 mL conical tube and left in an ultrasonic bath until completely dissolved, for approximately 10 min. The solution was then moved to a 50 mL beaker and left to stir at 100 rpm overnight to ensure complete evaporation of the solvents. The resulting thin film was rehydrated using 5 mL of PBS (pH 7.4) for 2 h. Residual amounts of methanol and chloroform may be unnecessarily toxic to off-target cells, and therefore these solvents had to be completely removed. The nano-vesicles were washed by placing the solution in an ultracentrifuge and were spun at 15,000 rpm for 1 h. The supernatant was replaced with PBS, vortexed until dispersed, and the nano-vesicles were then washed again using the same process for a second time. Finally, the formed vesicular dispersion was sonicated for 6 min to de-clump the mass of lipids, and to ensure the nano-vesicles retained similar sizes. Various ratios of miconazole, sodium oleate, and cholesterol (Table 1) were prepared to determine ideal amounts to maximize entrapment efficiency [19]. All samples were produced (at least) in triplicate. All experiments were performed (at least) in triplicate.

### 2.3. Determination of Vesicle Size, Polydispersity Index, and Zeta Potential

Vesicle size and polydispersity index were determined by using dynamic light scattering (DLS) and zeta potential was determined by electrophoretic mobility [20]. In total, 100 µL of nano-vesicles were diluted in 10 mL of de-ionized water and measurements were taken using a Malvern Zetasizer (Nano series ZS90, Malvern, Worchestershire, UK) at 25 °C.

### 2.4. Determination of Entrapment Efficiency

The entrapment efficiency (EE) of the formulations was determined using an ultracentrifugation method [21,22] using an Optima XPN-90 Ultracentrifuge (Beckman Coulter Life Sciences, Indianapolis, IN, USA) at 15,000 rpm for 4 h at 4 °C. After centrifugation, the supernatant was discarded to remove any unentrapped miconazole. In total, 10 mL of methanol was added to the lipid precipitate, and then sonicated in a bath for 1 h, and left for 12 h in a shaking water bath (25 °C at 100 rpm) to completely extract the entrapped miconazole. The resultant solution was centrifuged at 15,000 rpm for 1 h at 4 °C to separate the methanol lipid layer. The supernatant was then diluted, and the concentration of miconazole was determined using a Shimadzu UV-1800 spectrophotometer (Shimadzu Corporation, Kyoto, Japan), and the encapsulation efficiency was determined by using the following formula:

(1) $$Entrapment\;Efficiency=\frac{Miconazole\;remaining\;in\;vesicles}{Initial\;miconazole}\times 100$$

### 2.5. Determination of Viscosity

Viscosity testing was determined by using a fungilab Viscolead One (Fungilab, Hauppuage NY, USA) following the manufacturer’s instructions for a 200 mL sample [23]. The sample was placed in a cold-water bath and then tested at 5 °C, 24 °C, and 37 °C. These temperatures were chosen to determine if there would be significant changes in the formulation viscosity when at temperatures relating to cold storage, ambient temperature, and the average temperature of human skin.

### 2.6. In Vitro Permeation Study

The experiment was approved by Human Research Subject (IRB) Ethic Committee of the University of Texas at El Paso (Approval number 1193335, 25 January 2019). Skin preparation studies were performed using skin harvested from two human sources (Table 2) provided by the ZenBio corporation (ZenBio, East Triangle Park, NC, USA) listed below.

Various skin sources were used to determine any potential discrepancies in permeation due to sex or ethnicity, although few were expected [24]. Skin samples were stored in −20 °C until needed, in which case they were thawed for 24 h in 4 °C and then left to equilibrate at 24 °C for 20 min, then lightly shaved to remove any hair, if any existed. The skin was then soaked in 150 mL of PBS (pH 7.4) for 20 min for rehydration. The skin was then cut into squares of approximately 0.7 inch × 0.7 inch to fit into the ILC07 automated flow-through system (PermeGear Inc., Hellertown, PA, USA). The skin was then placed individually in one of the seven donor/receptor compartments.

These receptor compartments were locked in place with a small lower chamber that pumped PBS (pH 7.4) directly underneath the skin at 37 °C at a flow rate of 4 mL/h to simulate typical flow conditions of the human body [25]. The pump used was a multi-channel peristaltic pump IPC (Ismatec, Zurich, Switzerland) and the heating source was a Julabo BC4 circulating water bath (Julabo Pumps, Seelbach, Germany). The liquid that flowed through the chambers was collected in 20 mL scintillation vials, and changed at various time points (0.5, 1, 2, 3, 4, 5, 6, 7, 8, 12, 16, 20 and 24 h) to determine how much drug penetrated the final skin layer and could potentially enter the bloodstream. This process is presented in Figure 4A.

### 2.7. Separation of the SC Layer

In order to quantify the amount of drug left in the skin, the stratum corneum layer of skin was removed from the inner layers using a tape strip and scrape method [26]. The skin was carefully detached from the cells and placed on a flat metal surface. The skin was gently dabbed to remove excess formulation, and a piece of scotch tape was pressed against the skin to ensure maximum adherence. This strip was removed with light force and discarded. An additional piece of tape was placed on the skin, and the SC was then forcefully ripped from the other layers quickly in a single pull. This process was repeated 10 times. Additionally, a surgical scalpel was used to carefully scrape away and collect any leftover SC left after the tape stripping. The tape strips, along with the SC, were collected in conical tubes and dissolved in 10 mL of methanol.

The remaining dermal layers were cut with surgical scissors into the smallest pieces mechanically possible and placed into their own respective conical tubes. Each tube was then filled with 10 mL of methanol and sonicated for 30 min. The tubes were left overnight to soak at 4 °C. Finally, the solution was centrifuged at 15,000 rpm for 30 min at 4 °C and the supernatant was collected for drug quantification by UV-Vis spectrophotometer.

### 2.8. Fungal Assay

The fungal organisms used in this study were *Cryptococcus neoformans*. The fungus and fungal study were both provided and conducted in Dr. Luis Martinez’s laboratory in the department of Biology, University of Texas at El Paso. To evaluate the efficacy of miconazole–nano-vesicles, *Cryptococcus neoformans* [27], a powerful and somewhat resilient fungus, was cultured on a sabouraud dextrose agar plated petri dish [28]. To replicate a statistically significant result, a total of 32 plates with four treatments were prepared (*N* = 8 for each treatment). Using a Bunsen burner, the loop/needle were sterilized. After the fungus was successfully plated, the four treatments were plated with the fungus with PBS, 1% miconazole drug, 1% miconazole nanoparticles (NPs) and 2% miconazole nano-vesicle, respectively. The miconazole formulations were placed in four different spots in each petri dish with different treatments. A drop of the formulation (about 0.01 mL) was then placed in the cavity slide and was incubated for 25 ± 2 °C in a moist chamber to maintain proper humidity. Eight replicates were maintained for each treatment including the control. Each petri dish was examined for 48 h by observing the space without fungus between the drug and fungal colony. After 48 h, we took picture of each petri dish to measure the total inhibition caused by the miconazole formulations. The total inhibition area within a certain selected area in the petri dish was measured using ImageJ software (Version# 1.8.0_172) by NIH and using CAD software [29]. The inhibition areas for the various miconazole formulations were compared with the saline-treated petri dish and quantitatively analyzed heard-to-head. The ImageJ calculation is shown in Figure 5.

### 2.9. In Vitro Cytotoxicity/Biosafety

Human dermal fibroblast (HDF) cells were purchased from ATCC (Manassas, VA, USA). HDF cells were routinely cultured in Dulbecco’s Modified Eagle Medium (DMEM) supplement with 10% Fetal Bovine Serum (FBS) and 0.1% antibiotics (100 U/mL penicillin, 10 µg/mL streptomycin) in a humidified incubator with 5% carbon dioxide at 37 °C. When cell confluence reached 80%, they were incubated 5 × 10^3^ cell/well in a 96-well plate for 24 h. In total, 200 µL of medium was added in each well. Five different treatments were prepared with medium only (without cells), PBS, 1% miconazole- nano-vesicle nanoparticles (MUNP), 2% MUNP and 1% miconazole drug. Cells were prepared according to different time points starting from 1, 2, 4, 8 and 12 h respectively. The Vybrant^®^ 3-(4,5-dimethylthiazol-2-yl)-2,5-diphenyltetrazolium bromide (MTT) cell proliferation assay kit by Invitrogen, Thermo Scientific, was used according to the required specifications. The MTT cytotoxicity assay quick protocol [30,31] was also used according to the manufacturer’s specified instructions. In brief, 12 mM of 3-(4,5-dimethylthiazol-2-yl)-2,5-diphenyltetrazolium bromide (MTT) stock solution (MW = 414) was obtained by adding 1 mL of sterile PBS to one 5 mg vial of MTT; dimethyl sulfoxide (DMSO) was also used in this protocol. First, the medium was removed and replaced with 100 µL of fresh culture medium. Then, 10 µL of the 12 mM MTT stock solution was added to each well with 100 µL and incubated at 37 °C for 4 h. After labeling the cells with MTT, as described above, all but 25 µL of medium was removed from the wells. Then, 50 µL of DMSO was added to each well and mixed thoroughly with a pipette. After incubation for 10 min at 37 °C, absorbance was read at 540 nm using UV spectrophotometry Synergy 4 (BioTek, USA). Each sample was tested in triplicate. The untreated cells that were incubated with PBS were used as negative controls.

## 3. Results and Discussion

### 3.1. Preparation and Characterization

Various ratios of miconazole, cholesterol, and sodium oleate were evaluated to determine the ideal size, polydispersity, zeta potential, and encapsulation efficiency. The most ideal formulation was used for further study.

**Figure 2:**
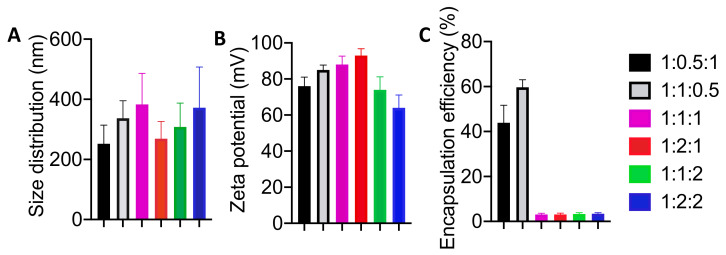
Size, zeta potential, and encapsulation efficiency amongst the various ratios of miconazole:cholesterol:sodium oleate. (**A**) Size distribution and (**B**) zeta potential remained relatively similar, however high variation between vesicles eliminated consideration for 1:1:1 and 1:2:2 due to an inordinate number of vesicles stretching beyond the nano-range. (**C**) Encapsulation efficiency amongst the various ratios eliminated several formulations from consideration due to poor performance in relation to drug delivery, while two ratios conversely displayed exceptional loading potential. All data are presented as mean ± SE (*n* = 3).

Miconazole sizes were all found to be within the range of less than 400 nm (Figure 2), which, according to previous studies, would prove to be ideal for passing through the stratum corneum to deliver miconazole to the deeper layers within the skin [32]. Standard deviation remained relatively consistent, allowing any of the formulation ratios to be considered for additional evaluation. These results were to be expected due to the identical preparation methods and would not be the main determinant for drug delivery. Similar findings were observed in previous studies with clotrimazole and diclofenac [33].

Like the size evaluation, the measurement of the electrokinetic zeta potential proved to be equally acceptable across all ranges, displaying excellent stability. This is a key indicator of colloidal dispersions, and offers a promising view of the formulation stability [34].

The encapsulation efficiency is a prime indicator that the formulations displayed key differences (Figure 2), and eliminated the concept of allowing certain ratios to be considered for further study as drug carriers [35]. Four of the formulations displayed abysmal encapsulation efficiency, all approximately at 3%, making them relatively inadequate for any method of efficient drug delivery. Two of the formulations (1:1:0.5 and 1:0.5:1) displayed high rates of encapsulation; however, the 1:1:0.5 formulation offered the highest encapsulation efficiency (~60%) with a lower standard deviation than its counterpart. Due to these advantages, the 1:1:0.5 formulation of miconazole:cholesterol:sodium oleate was determined to be the best ratio combination to use as a transdermal drug delivery vehicle for miconazole, and was used for all further studies beyond this point [36].

**Figure 3:**
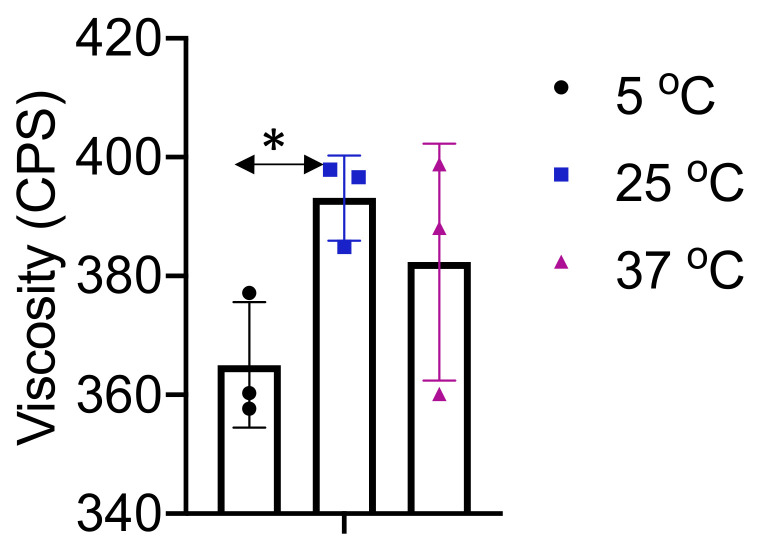
Viscosity testing was performed at various temperatures to determine the optimal temperature during administration, as well as to see if any major change occurred at temperatures similar to contact with human skin. Viscosity remained relatively constant throughout the spectrum, remaining between 355 and 405 centipoise (CPS). All data are presented as mean ± SE (*n* = 3). *p < 0.05 compared to 25 °C.

Viscosity studies provided an insight into the changes at various temperatures (Figure 3). Viscosity remained within 355–405 CPS, in between all temperature changes, and performed as expected with very little difference in viscosity. The various materials used in the formulation may have impacted the formulation slightly with the temperature changes, but all maintained a very liquid state throughout. With the abundance of PBS present in the formulation, the application would be expected to display little to no difference regardless of conventional temperatures while in cold storage, or after being left at room temperature.

### 3.2. In Vitro Diffusion

**Figure 4:**
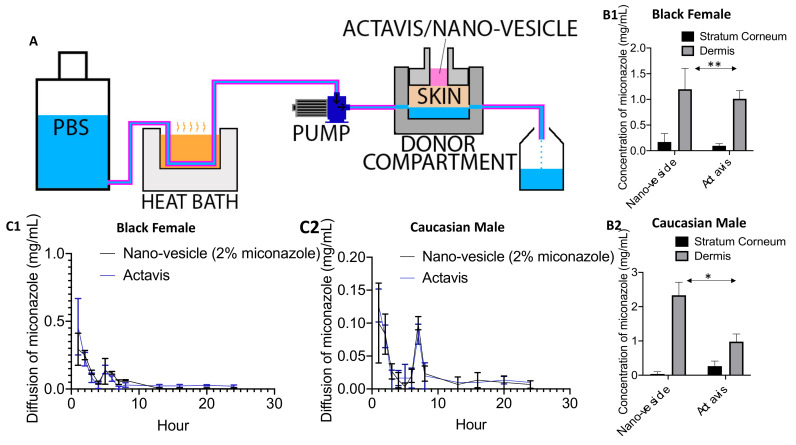
(**A**) Diagram of the PermeGear Franz diffusion cell system. PBS is warmed to 37 °C before being pumped into the donor compartment, underneath the skin, and finally resides in the scintillation vial, which was changed hourly. (**B1**,**B2**) UV-Vis comparing the amount of miconazole located within the stratum corneum and lower dermal layers after 24 h in the PermeGear diffusion system. (**C1**,**C2**) Hourly readings of the PBS collected scintillation vials, identifying the amount of drug that may pass completely through the skin and into the bloodstream within 24 h. All data are presented as mean ± SE (*n* = 6). *p < 0.05; **p < 0.01 compared with the Actavis.

In vitro diffusion studies supported the notion that large amounts of miconazole stayed within the dermal layers after 24 h (Figure 4B1,B2), significantly more when compared to the Actavis market cream [37]. This supports the notion that nano-vesicles are more effective in delivering this insoluble drug to the target site than the conventional delivery methods currently being marketed [33]. These higher quantities can offer significantly more therapy than their marketed counterparts. Higher amounts of miconazole were found in the younger Caucasian male. Younger skin tends to display higher levels of elasticity and retains significantly more moisture than aging skin. This may be the reason why a significantly larger amount of miconazole was able to penetrate and reside in the younger skin (Figure 4B2).

Analyses of the PBS collected hourly showed that very little miconazole passed beyond the skin (Figure 4C1,C2), indicating that most of the drug achieved a prolonged presence in the target site. Like other studies, this again supports the notion that lipid-based nanoparticles greatly enhance the penetration of molecules through the skin, while staying within the target site instead of being metabolized by the body. These promising results signify the potential to greatly increase the current levels of drug delivery through the skin, while avoiding systemic circulation and elimination [38].

### 3.3. Anti-Fungal Study

**Figure 5:**
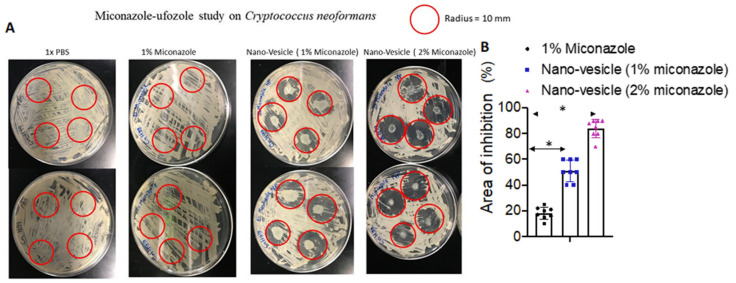
Fungal growth inhibition using free miconazole, as well as nano-vesicles at 1% and 2% miconazole. PBS was used as a control. (**A**) Significantly higher levels of inhibition were expressed using nano-vesicles as opposed to free miconazole, supporting the notion that nano-vesicles act as an inhibition enhancer. (**B**) Around 20% and 40% total fungal colonies were inhibited after 48 h with the miconazole nano-vesicles 1% and 2% treatment compared to PBS treated petri dishes, respectively. All data are presented as mean ± SE (*n* = 8). *p < 0.05 compared with the Actavis.

In Figure 5, the summarized effectiveness of miconazole nano-vesicles has been displayed, showing that both 1% and 2% miconazole containing nano-vesicles were able to kill higher fungal colonies compared with 1% miconazole-vesicles, as well as the drug itself. This clear visual experiment shows that miconazole nano-vesicles can inhibit fungal colonies at a rate of 20% and 40% (Figure 5) with 1% and 2% miconazole nano-vesicle treatment, respectively. The maximum zone of inhibition by 1% and 2% miconazole nano-vesicles was calculated as 15.54 ± 0.092 mm^2^ and 11.74 ± 0.148 mm^2^, respectively when compared with 1% of the drug and the negative control (PBS) with 19.315 ± 0.015 mm^2^ and 19.64 ± 0.0 mm^2^, respectively (Figure 5). The projected efficiency of nano-vesicles to inhibit fungal colony germination compared with a negative control and 1% of the standalone drug in terms of total area is also exhibited. The presence of a larger inhibition zone in the fungal colonies [39] clearly indicates the higher level of efficiency of nano-vesicles over direct drug application.

### 3.4. Cytotoxicity Biosafety Study

**Figure 6:**
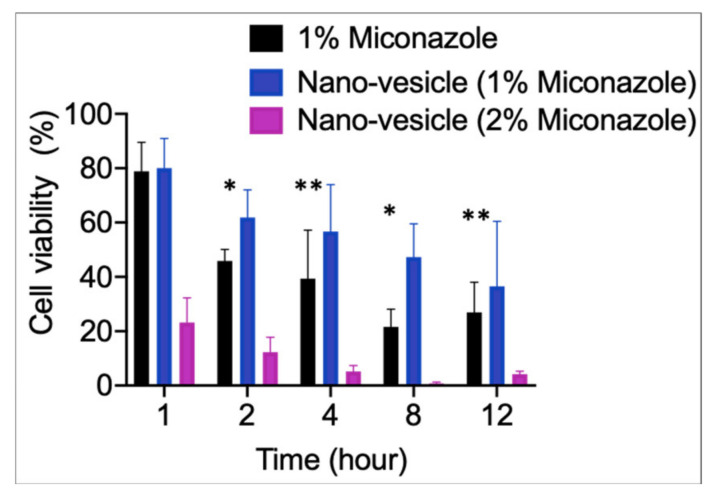
Cell viability at varying time points. The 2% miconazole nano-vesicles displayed lower cell viability at all time points when compared with 1% miconazole nano-vesicle and 1% free miconazole. All data are presented as mean ± SE (*n* = 9). *p < 0.05 and **p < 0.5 for nano-vesicle compared to (Actavis) of 1% miconazole.

As seen in Figure 6, nano-vesicles loaded with 1% miconazole displayed higher cell viability than the free miconazole counterparts. However, when nano-vesicles loaded with 2% miconazole were introduced to the cells, significantly lower cell viability was observed. This may be due to the overabundance of miconazole being present; however, the amount of drug present is still well below the expected LD50. Alternatively, this may be due to the lower drug loading capability of the miconazole due to the 2% formulation essentially being a concentrated combination of nano-vesicle and miconazole. Both of these hypotheses lend credence to the notion that further observation is required for the application of this transdermal drug delivery method [40]. This data also suggest that 1% miconazole nano-vesicles are a much safer alternative to 1% free miconazole drug treatments [41].

## 4. Conclusions

The increased bioavailability of conventional over the counter (OTC) drugs can greatly enhance their efficacy simply and effectively. In this study, we characterized a novel miconazole nano-vesicle carrier, optimized the formulation (100 mg miconazole, 100 mg cholesterol, 50 mg sodium oleate) in (approximately) a 400 nm diameter lipid carrier and, at the same time, achieved a high entrapment efficiency (60%). In vitro permeation studies display significant targeted transdermal drug delivery to the site of infection in higher amounts than the conventional marketed cream. Antifungal studies show that the current formulation greatly enhances the efficacy of antifungal activity when compared to standalone miconazole. These enhancements have the potential to revitalize the efficacy and conventional use of miconazole, even at the over the counter market level, and therefore require further development to demonstrate and understand its efficacy in an animal model. This novel miconazole delivery system offers the potential to transform the current market, revolutionizing conventional medicines and offering a new wave of enhanced revitalized treatments using already understood conventional drugs and therapies.

## Figures and Tables

**Table 1:** Display of the various ratios of miconazole, cholesterol, and sodium oleate used in each formulation. Miconazole was used as the determinant anchor to maintain consistency with testing, and to offer a support when comparing with Actavis brand marketed cream.

Formulations	Miconazole (mg)	Cholesterol (mg)	Sodium Oleate (mg)	Miconazole Loading Efficiency (%)	Size Distribution (nm)	Zeta Potential (mV)
1:0.5:1	100	50	100	43.9 ± 7.8	252 ± 62	76 ± 5
1:1:0.5	100	100	50	59.7 ± 3.3	337 ± 58	85 ± 3
1:1:1	100	100	100	3.1 ± 0.5	383 ± 103	88 ± 5
1:2:1	100	200	100	3.1 ± 0.6	269 ± 57	93 ± 4
1:1:2	100	100	200	3.3 ± 0.6	308 ±79	74 ± 7
1:2:2	100	200	200	3.4 ± 0.5	372 ± 135	64 ± 7

**Table 2:** Skin samples used for diffusion studies. Samples were taken from the same location (abdomen) and with similar scarring (severe); however, the age, gender, and ethnicity were different between the samples. This was done purposely to determine if the intake of miconazole vesicles would act differently when alternative factors were introduced, and to compare these potential differences with those of a marketed cream (Actavis).

Ethnicity	Gender	Age	BMI	Stretch Marks	Location
Caucasian	Male	24	29.9	Severe	Abdomen
African/Black	Female	43	26.9	Severe	Abdomen
